# Fabrication of Superhydrophobic Mg/Al Layered Double Hydroxide (LDH) Coatings on Medium Density Fiberboards (MDFs) with Flame Retardancy

**DOI:** 10.3390/ma11071113

**Published:** 2018-06-29

**Authors:** Zhe Wang, Xiaoping Shen, Temeng Qian, Kang Xu, Qingfeng Sun, Chunde Jin

**Affiliations:** 1 School of Engineering, Zhejiang A&F University, Hangzhou 311300, China; donjade@163.com (Z.W.); sxp1031@hotmail.com (X.S.); teemeeng@163.com (T.Q.); 2 Zhejiang Academy of Forestry, Hangzhou 310023, China; xkang86@126.com

**Keywords:** medium density fiberboard, Mg/Al LDH, FDTS, superhydrophobicity, flame retardancy

## Abstract

The hydrophilicity and flammability of fiberboards have limited their real-life applications. In this study, a facile strategy for preparing the multifunctional coatings with superhydrophobicity and flame retardancy on medium density fiberboards (MDFs) has been investigated. The superhydrophobic and flame-retardant coating on the MDF surface was obtained by depositing polydimethylsiloxane (PDMS) and 1H, 1H, 2H, 2H-perfluorodecyltrichlorosilane (FDTS)-modified Mg/Al layered double hydroxide (LDH) particles step by step. The as-prepared coating exhibited superhydrophobic properties with a water contact angle (WCA) of ~155° and good self-cleaning properties. Furthermore, the limiting oxygen index (LOI) value of the superhydrophobic MDFs increased by 60.4% as compared to that of the pristine MDFs, showing improved flame retardancy. The peak heat release rate (PHRR) and total heat release (THR) of MDFs decreased after coating with PDMS@FDTS-Mg/Al LDH, suggesting that the superhydrophobic coating decreased the fire growth speed and risk of fire hazard of MDFs. This coating with multiple functions opens a new avenue for the protection and functionalization of MDFs.

## 1. Introduction

Fiberboards have been widely applied in various fields, for example in furniture and interior decoration materials. This is due to their abundance, low cost, and easy machinability [1,2]. In general, the fiberboards exhibit hydrophilic, hygroscopic, and flammable properties during application due to the existence of abundant hydroxyl groups in the wood fiber surface, their porous structure, and organic nature [3,4,5]. This sensitivity to humidity changes and flame usually results in a decrease in the service life of fiberboards and a threat to the human safety. Therefore, hydrophobization treatments and improvements of flame retardancy are exceedingly important for the hydrophilic and flammable fiberboards in order to prolong service life and increase safety.

Adding wax is considered as the most common method for improving the dimensional stability of fiberboards during manufacture due to the resulting hydrophobicity [6,7]. However, this results in a decrease in the mechanical properties of the fiberboards [8,9]. On the other hand, this method is not able to fundamentally solve the problem of deformation after absorbing water. Inspired by nature, superhydrophobic surfaces with a hierarchical structure at the micro- and nano-scale and low surface energy have been fabricated on a variety of substrates [10,11,12]. In recent years, superhydrophobic coatings have been successfully prepared on wood-based materials by varied methods [10,13,14,15]. Wang firstly synthesized silica nanoparticles on poplar wood surface via a sol-gel process and then carried out hydrophobic treatment using 1H, 1H, 2H, 2H-perfluoroalkyltriethoxysilanes to obtain a superhydrophobic coating [16]. Tu reported a method for fabrication of a durable and self-healing superhydrophobic coating on Chinese fir wood. This coating was obtained by two steps, including coating a polydimethylsiloxane (PDMS) film and then spraying a mixture of perfluoroalkyl methacrylic copolymer and TiO_2_ nanoparticles [17]. Kong prepared superhydrophobic and flame-retardant ZnO coating on wood by a hydrothermal process. The ZnO nanorod arrays were firstly fabricated on wood via a hydrothermal method to generate surface roughness and then immersed in stearic acid solution to obtain low surface energy [18]. Chen reported a superhydrophobic coating with superparamagnetic property by a soft lithography method [19]. These methods for fabrication of superhydrophobic surfaces on wood-based materials also offer a new path for improving hydrophobicity of medium density fiberboards (MDFs).

Furthermore, traditional flame-retardant methods of fiberboards involve mixing flame retardants with adhesives or fibers before hot pressing progress. At early stage, halogen-containing compounds are used as the most common flame retardants. However, the leakage of hazardous flame retardants would have a bad influence on the environment and human health [20]. In order to overcome these disadvantages, some inorganic flame retardants are used, such as nitrogen- or phosphorus-containing compounds, Al(OH)_3_ and clay [21,22,23], and so on. Layered double hydroxides (LDHs) are composed of positively-charged metal hydroxide layers, interlayer anions, and water. The general formula for hydrotalcites is [M^2+^_1−x_M^3+^_x_(OH)_2_]^x+^[A^p−^_x/p_]^x+^ · mH_2_O, where M^2+^ and M^3+^ are a metallic bivalent cation and a metallic trivalent cation, respectively, and A^p−^ is an interlayer anion [24,25,26]. Among them, Mg/Al LDHs have attracted widespread interests due to their strong flame retardancy resulting from the synergistic effect of Al(OH)_3_ and Mg(OH)_2_ [27,28]. Wang reported a facile method to fabricate Co_2_/Al–LDH polypropylene composites with improved flame retardancy [29]. Kuila fabricated a rubber/DS^−^–modified LDH composite with enhanced flame-retardant properties [30]. Guo prepared a superhydrophobic Mg/Al LDH coating with flame-retardant and smoke-suppression properties on wood via a two-step process. Mg/Al LDHs were firstly fabricated on wood via a hydrothermal method and then immersed in trimethoxy(1H,1H,2H,2H-heptadecafluorodecyl) silane to generate superhydrophobicity [31]. However, the fabrication process of the above Mg/Al LDH coating is complex. A facile fabrication process of Mg/Al LDH coatings is urgently needed. Moreover, as far as we know, up to now there has been no report on the preparation of superhydrophobic Mg/Al LDH surfaces with flame retardancy on MDFs.

Herein, we report a facile method to prepare superhydrophobic coating with flame retardancy on MDF via brushing coating of PDMS following by deposition of 1H, 1H, 2H, 2H-perfluorodecyltrichlorosilane (FDTS)-modified Mg/Al LDHs. In this coating system, Mg/Al LDH act as hierarchical roughness materials and FDTS functions as low surface energy film. PDMS serves as adhesive to anchor the modified Mg/Al LDH on the MDF surface. Additionally, due to the coated Mg/Al LDHs, the superhydrophobic coatings endow the MDF with improved flame retardancy. It is believed that the resulting multifunctional superhydrophobic coating can provide a new strategy for the protection and functionalization of MDFs.

## 2. Materials and Methods

### 2.1. Materials

Wood fibers were provided by Zhejiang Great World Group (Ningbo, China). Chitosan was supplied by Macklin Biochemical Co., Ltd. (Shanghai, China). Glutaraldehyde (25 wt %) and absolute ethanol were provided by Sinopharm Chemical Reagent Co., Ltd. (Shanghai, China). 1H, 1H, 2H, 2H-perfluorodecyltrichlorosilane (FDTS) was purchased from Aladdin (Shanghai, China). Mg/Al layered double hydroxides (Mg_4_Al_2_(OH)_12_CO_3_·3H_2_O) were purchased from the Usolf chemical technology company (Qingdao, China). Polydimethylsiloxane (PDMS) and its curing agent were supplied by Dow Corning Company (Midland, MI, USA).

### 2.2. Preparation of Medium Density Fiberboards

Medium density fiberboards were prepared according to our previous studies [5]. In a typical process, a mixture of 2% (*w*/*v*) was obtained by dissolving chitosan in an acetic acid solution of 1.5% (*w*/*v*) at room temperature for 2 h under stirring. Secondly, 20 wt % (*w*/*w*, to chitosan) glutaraldehyde was dropwise injected into the chitosan mixture with continuous agitation until the formation of chitosan hydrogel. After that, the wood fibers were mixed with chitosan hydrogel following a mass ratio of 5.0:100 (chitosan to wood fibers). Finally, the blended fibers were hot pressed at 180 °C, 4.5 MPa to form a board with a size of 200 mm × 200 mm × 8 mm. The target density of fiberboard was 0.83 g/cm^3^.

### 2.3. Preparation of FDTS-Modified Mg/Al LDH

Firstly, 1.0 g of Mg/Al LDHs was added into 20 mL absolute ethanol containing 1% FDTS (*v*/*v*) and dispersed by ultrasonic treatment for 30 min. After that, the above suspension was stirred for 24 h at room temperature. Then, the resultant suspension was centrifuged and washed several times with absolute ethanol. Finally, the FDTS-Mg/Al LDHs were obtained after dried at 60 °C.

### 2.4. Preparation of PDMS@FDTS-Mg/Al LDH Coating on MDF

**Figure 1:**
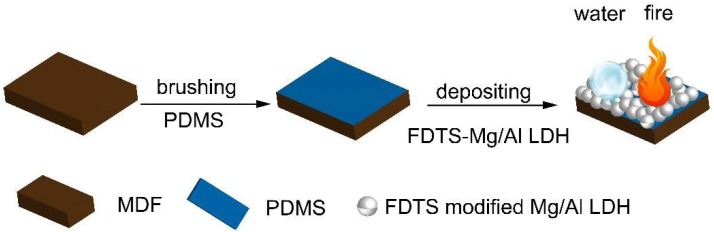
Schematic illustration of the procedure to fabricate PDMS@FDTS-Mg/Al LDH coating on the MDF surface. PDMS: Polydimethylsiloxane; FDTS: 1H, 1H, 2H, 2H-perfluorodecyltrichlorosilane; LDH: Layered double hydroxide; MDF: Medium density fiberboard.

Figure 1 presents the fabrication process of the PDMS@FDTS-Mg/Al LDH coating. Firstly, a mixture containing PDMS and its curing agent was prepared at room temperature following a mass ratio of 10:1. Afterwards, the PDMS mixture was uniformly covered on the MDF surface using a brush. After that, the MDF surface coated with PDMS was further covered by the as-prepared FDTS-Mg/Al LDH particles. Finally, the MDF surface with superhydrophobic and flame-retardant properties was obtained after drying at 70 °C for 5 h.

### 2.5. Characterization

The surface chemical compositions measurements of the pristine and modified Mg/Al LDH particles were performed using X-ray photoelectron spectroscopy (XPS, Thermo ESCALAB 250XI, Thermo, Waltham, MA, USA). The chemical group changes of Mg/Al LDH before and after modification were recorded by Fourier transform infrared spectroscopy (FTIR, iS10, Nicolet, Waltham, MA, USA) using the KBr pellet method. The crystalline structures of the pristine and modified Mg/Al LDH particles were observed by X-ray diffraction (XRD, XRD-6000, Shimadzu, Kyoto, Japan) with Cu Kα radiation at 40 kV and 30 mA, λ = 0.1540 nm. Surface morphology of MDF before and after coating treatment were observed using scanning electron microscopy (SEM, Hitachi SU8010, Hitachi, Tokyo, Japan). Prior to the observation, thin gold films were sputtered on all samples by using a low-conductivity sputtering coater. Surface chemical composition mapping images of MDF coated with PDMS@ FDTS-Mg/Al LDH were obtained by energy dispersive X-ray spectrometry (EDS). The water contact angles (WCAs) of MDF before and after coating treatment were measured using an OCA100 contact angle test system (DataPhysics, Stuttgart, Germany) at room temperature. A 4 μL water drop was used to measure the WCA and the average values of WCA were obtained by five measurements at different positions. Limiting oxygen index (LOI) values of uncoated and coated MDF with a size of 150 mm × 10 mm × 8 mm were tested using a JF-5 oxygen index instrument. Combustion parameters of MDFs (100 mm × 100 mm × 8 mm) before and after coating were tested via a cone calorimeter (FTT Company, Derby, UK) using 50 kW m^−2^ irradiance.

## 3. Results and Discussion

### 3.1. XPS Analysis

**Figure 2:**
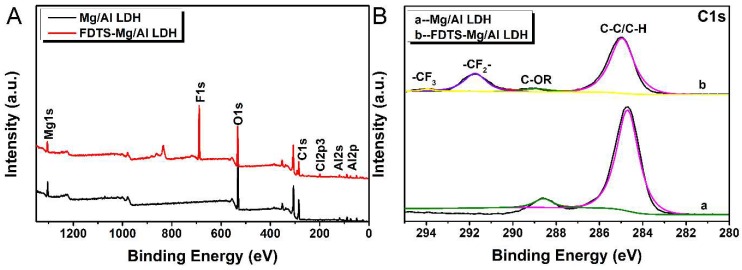
XPS spectra of Mg-Al LDH and FDTS-modified Mg-Al LDH: (**A**) survey spectra; (**B**) C1s.

XPS spectra was used to evaluate the surface chemical compositions of Mg/Al LDH before and after modification. Figure 2A presents the XPS survey spectra of Mg/Al LDH and FDTS modified Mg/Al LDH. As shown in Figure 2A, for both Mg/Al LDH and FDTS-modified Mg/Al LDH peaks appeared at 74 eV, 120 eV, 285 eV, 533 eV, and 1305 eV belonging to Al2p, Al2s, C1s, O1s, and Mg1s [31], respectively. However, two additional peaks can be observed in FDTS-modified Mg/Al LDH at 199 eV and 689 eV assigned to Cl2p3 and F1s, respectively [32]. Figure 2B shows the C1s high-resolution spectrum of Mg/Al LDH and FDTS-modified Mg/Al LDH. The fitting peaks of unmodified and modified Mg/Al LDH at 284.9 eV and 285.5 eV were assigned to C–C or C–H and C–OR. The C–C or C–H bond of unmodified and modified Mg/Al LDH may be due to carbon contamination. Furthermore, the C–C or C–H bond of modified Mg/Al LDH also could result from FDTS. Additionally, the modified Mg/Al LDH displayed two additional fitting peaks at 291.9 eV and 294.2 eV belonging to –CF_2_– and –CF_3_ [32]. These indicated that long-chain FDTS was successfully attached to Mg/Al LDH surfaces.

### 3.2. FTIR and XRD Analysis

**Figure 3:**
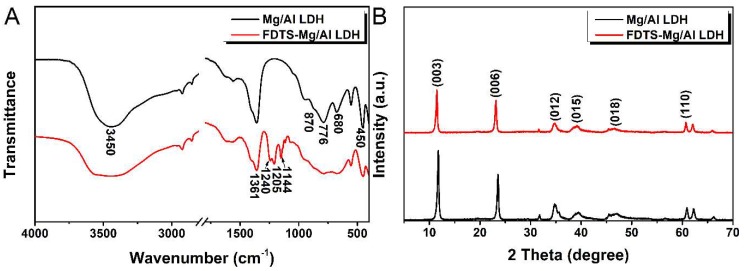
FTIR spectra (**A**) and X-ray diffraction patterns (**B**) of Mg-Al LDH and FDTS-modified Mg/Al LDH.

In order to further investigate the modifications of Mg/Al LDH, FTIR spectra was carried out on Mg/Al LDH and FDTS modified Mg/Al LDH (Figure 3A). As shown in Figure 3A, the absorption peaks appeared at 3450 cm^−1^ ascribed to –OH stretching vibrations of bare Mg/Al LDH. After modified by FDTS, it can be clearly observed that the broader absorption peaks appeared at 3403 cm^−1^ in the spectra of modified Mg/Al LDH, indicating that the hydrogen bond interaction occurred between FDTS and Mg/Al LDH. When FDTS was added to ethanol, a hydrolysis reaction of FDTS occurred. Si–OH groups can be obtained due to the hydrolysis of the three Si–Cl groups of FDTS [32]. Thus, it can induce hydrogen bond formation between Si–OH groups and –OH groups of Mg/Al LDH. Furthermore, a strong peak was observed at 1361 cm^−1^ attributed to *ν*_3_ vibration of CO_3_^2−^ from the interlayer anion [33]. For both Mg/Al LDH and modified Mg/Al LDH, absorption peaks appeared at 450, 680, 776 and 870 cm^−1^, attributed to Mg–O and Al–O vibrations [34,35,36]. Furthermore, several additional peaks appeared at 1240, 1205, and 1144 cm^−1^ belonging to CF_2_ asymmetric stretching, FH_2_ symmetric stretching, and CF_2_ symmetric stretching in the spectra of modified Mg/Al LDH [37,38,39], which indicated that long-chain fluoroalkyl functionalized Mg/Al LDHs were obtained.

XRD measurements were used to investigate the crystalline structure changes of Mg/Al LDH before and after modification. Figure 3B shows the XRD patterns of Mg/Al LDH and FDTS modified Mg/Al LDH. As shown in Figure 3B, for Mg/Al LDH crystalline peaks appeared at 11.76°, 23.58°, 34.76°, 39.56°, 47.24°, and 60.94°, assigned to the diffraction of the (003), (006), (012), (015), (018) and (110) plane of quintinite-3R (Mg_4_Al_2_(OH)_12_CO_3_·3H_2_O) [26,40,41]. As shown in Table 1, after modification, (003), (006), (015), (018), (110) diffraction peaks shifted to lower angles. Meanwhile, their d values were higher than those of unmodified Mg/Al LDH. This may be due to the anion exchange reaction between FDTS and LDH, which led to the increase in the interlayer distance. The conceivable reaction formula can be seen as follows.

(1) $${\mathrm{Mg}}_{4}{\mathrm{Al}}_{2}{\left(\mathrm{OH}\right)}_{12}{\mathrm{CO}}_{3}\cdot 3H_{2}O\to {\mathrm{Mg}}_{4}{\mathrm{Al}}_{2}{\left(\mathrm{OH}\right)}_{12}{\mathrm{CO}}_{3}+3H_{2}O$$

(2) $$F_{17}C_{8}C_{2}H_{4}{\mathrm{SiCl}}_{3}\overset{\mathrm{hydrolysis}}{\to }F_{17}C_{8}C_{2}H_{4}\mathrm{Si}{\left(O^{-}\right)}_{3}+3H^{+}$$

(3) $${\mathrm{Mg}}_{4}{\mathrm{Al}}_{2}{\left(\mathrm{OH}\right)}_{12}{\mathrm{CO}}_{3}+F_{17}C_{8}C_{2}H_{4}\mathrm{Si}{\left(O^{-}\right)}_{3}\overset{\mathrm{ion}\;\mathrm{exchange}}{\to }F_{17}C_{8}C_{2}H_{4}\mathrm{Si}{\left(O^{-}\right)}_{3}-\mathrm{LDH}$$

**Figure 4:**
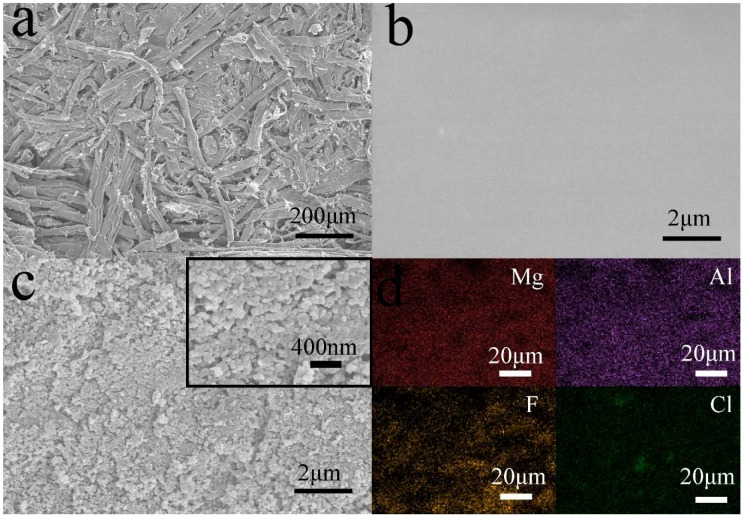
SEM images of MDFs (**a**); MDFs coated with PDMS (**b**); MDFs coated with PDMS@FDTS-Mg/Al LDH (**c**); EDS mapping images of MDFs coated with PDMS@FDTS-Mg/Al LDH (**d**).

The superhyrophobicity is commonly considered to be due to the synergistic effects of micro- and nano- hierarchical structures and low surface energy. Therefore, it is necessary to investigate the surface morphologies changes of MDFs before and after coating treatment. Figure 4 shows the SEM images of MDFs, MDFs coated with PDMS, and MDFs coated with PDMS@FDTS-Mg/Al LDH, as well as EDS mapping images of MDFs coated with PDMS@FDTS-Mg/Al LDH. As shown in Figure 4a, MDF surfaces exhibited a rough structure composed of hydrophilic wood fibers. After coated by PDMS (Figure 4b), the initial rough structures of MDF surfaces cannot be observed. It was clearly observed that a smooth surface was obtained, indicating that the PDMS film uniformly coated on the MDF surface. As shown in the SEM image of MDF coated with PDMS@FDTS-Mg/Al LDH (Figure 4c), FDTS-Mg/Al LDH particles were distributed on the MDF surface in a few micrometers. Meanwhile, it can be observed that nano-scale particles were also displayed on the MDF surface (Figure 4c insert). According to the Cassie and Baxter’s law, the decrease in wettability of solid material surfaces can be attributed to the increased area occupied by air between solid and liquid interface. Therefore, this micro- and nano-scale hierarchical structure was beneficial to create superhydrophobicity. The surface elemental composition distributions of MDF coated with PDMS@FDTS-Mg/Al LDH are shown in EDS mapping images. It can be seen that the elements of magnesium, aluminum, fluorine, and chlorine exhibited the uniform distribution on the MDF surfaces. This also demonstrated that the hydrophobic coatings with fluoroalkane groups were successfully covered on MDF surfaces.

**Figure 5:**
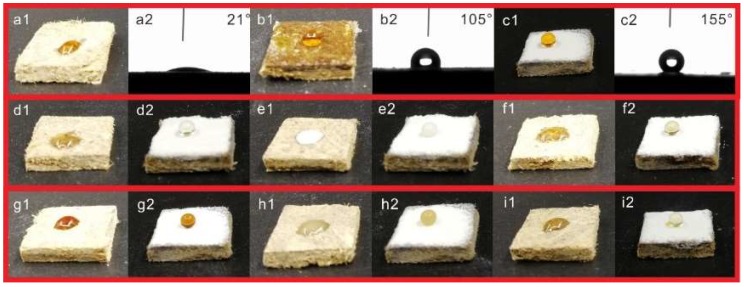
Digital photos and contact angle images of water on different surfaces: (**a**) MDFs; (**b**) MDFs coated with PDMS; (**c**) MDFs coated with PDMS@FDTS-Mg/Al LDH; Images of different liquids on the surface of MDFs (**d1**–**i1**) and MDFs coated with PDMS@ FDTS-Mg/Al LDH (**d2**–**i2**): (**d**) tea; (**e**) milk; (**f**) cola; (**g**) soy sauce; (**h**) coffee; (**i**) orange juice.

In order to evaluate the wettability changes before and after coating treatment, the contact angle tests were carried out. As shown in Figure 5a1,a2, the water droplet was promptly absorbed by MDF (WCA = 21°), indicating that the MDF surfaces were hydrophilic. As can be seen from Figure 5b1,b2, the water droplet exhibited a semispherical shape on MDF surfaces (WCA = 105°) after being coated with PDMS, which can be attributed to the hydrophobicity of PDMS. It was clearly observed that the near-spherical water droplet stood on the MDF coated with PDMS@FDTS-Mg/Al LDH (WCA = 155°), indicating the superhydrophobicity of PDMS@FDTS-Mg/Al LDH coatings. As common indoor decoration materials, MDFs may be attacked by common household liquids. Therefore, it was important to evaluate the domestic liquid repellency of the coatings. The digital images of tea, milk, orange juice, soy sauce, coffee and cola on the uncoated and coated MDFs are shown in Figure 5d–i. As shown in Figure 5d–i, the uncoated MDFs were immediately infiltrated by the above six liquids. In contrast, all the six liquid droplets stood on MDFs coated with PDMS@FDTS-Mg/Al LDH with a sphere, suggesting that this coating presented good domestic liquid repellency.

**Figure 6:**
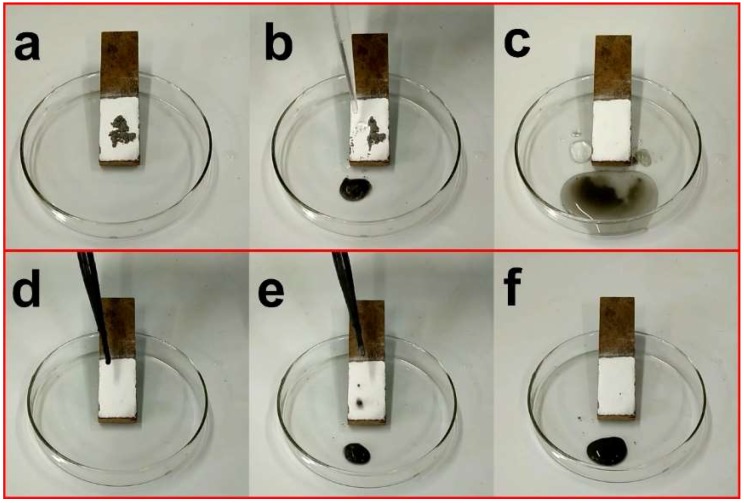
Self-cleaning properties of FDTS-modified Mg/Al LDH coatings. (**a**) Fly ashes on the PDMS@FDTS-Mg/Al LDH coating; (**b**) Fly ashes are taken away by water droplets ; (**c**) Clear coating after water droplets washing; (**d**) Original coating before muddy water washing; (**e**) Muddy water roll away from coating; (**f**) Clear coating after muddy water washing.

Furthermore, dust or particle contamination on the PDMS@FDTS-Mg/Al LDH coating cannot be avoided in daily life. Hence, it was also important to investigate the self-cleaning properties of PDMS@FDTS-Mg/Al LDH coating. Fly ashes as contamination source were deliberately positioned on the coating (Figure 6a). The water droplets can easily roll off from the coating and concurrently take away the fly ashes (Figure 6b). Finally, the initial white coating was left (Figure 6c). Moreover, muddy water was obtained by the mixture of fly ashes and water to evaluate the self-cleaning properties toward liquid contaminations. Likewise, the muddy water readily rolled away from the coating without traces (Figure 6d–f), indicating that the coating displayed a good self-cleaning property.

**Figure 7:**
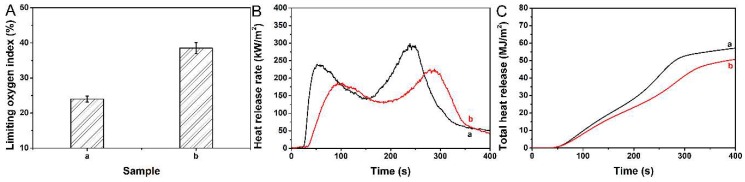
Limiting oxygen index (**A**), heat release rate (**B**) and total heat release (**C**) of MDFs and MDFs coated with PDMS@FDTS-Mg/Al LDH: a and b refer MDFs and MDFs coated with PDMS@FDTS-Mg/Al LDH.

As the most common parameters for investigating the difficulty of combustion, the LOI was used to evaluate the flame retardancy of MDFs coated with PDMS@FDTS-Mg/Al LDH. As shown in Figure 7A, the pristine MDFs were easily flammable, with a LOI value of 24.0. In contrast, the LOI value of MDFs coated with PDMS@FDTS-Mg/Al LDH increased to 38.5 due to the flame retardancy of Mg/Al LDH. This showed that the PDMS@FDTS-Mg/Al LDH coating improved the flame retardancy of MDF. The flame retardancy of MDF coated with PDMS@FDTS-Mg/Al LDH was further evaluated by the cone calorimeter test. The cone calorimeter test data of MDF and MDF coated with PDMS@FDTS-Mg/Al LDH are shown in Figure 7B,C and Table 2. The ignition time of MDF coated with PDMS@FDTS-Mg/Al LDH (42 s) was prolonged 21 s as compared to MDFs alone (21 s), indicating that it was harder to ignite MDF coated with PDMS@FDTS-Mg/Al LDH. As shown in Figure 7B, the peak heat release rate (PHRR) decreased from 298.8 kW/m^2^ (MDF) to 224.9 kW/m^2^ (MDF coated with PDMS@FDTS-Mg/Al LDH). Moreover, the fire growth rate (FIGRA) decreased from 1.3 kW/m^2^ s (MDF) to 0.8 kW/m^2^ s (MDFs coated with PDMS@FDTS-Mg/Al LDH). As shown in Figure 7C, the total heat release (THR) of MDFs coated with PDMS@FDTS-Mg/Al LDH decreased by 11.2% as compared to that of pristine MDFs. These also demonstrated that coating PDMS@FDTS-Mg/Al LDH was beneficial to reduce the risk of fire hazard.

**Figure 8:**
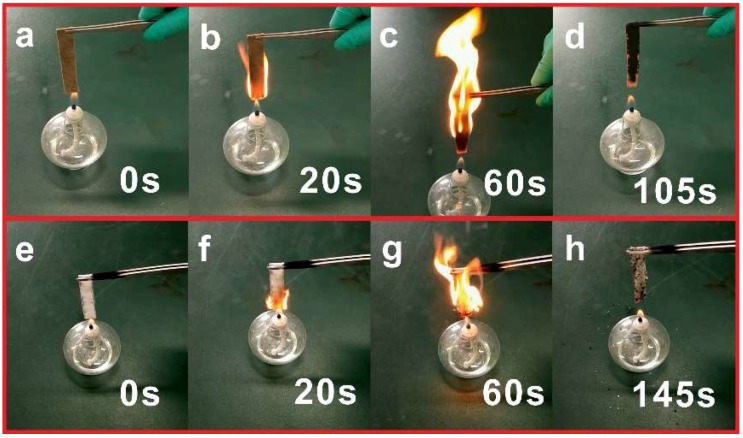
Combustion process of (**a**–**d**) MDFs; (**e**–**h**) MDFs coated with PDMS @FDTS-Mg/Al LDH.

In order to investigate the combustion process of MDF in practical applications, the MDFs and MDFs coated with PDMS@FDTS-Mg/Al LDH (100 mm × 15 mm × 8 mm) were burned above an alcohol burner (Figure 8). As shown in Figure 8b, half the area of the pristine MDFs was ignited after 20 s. It was clearly observed that the blazing fire spread to the top of the MDFs after 60 s (Figure 8c). MDFs burned out with a black ash after 105 s (Figure 8d). In contrast, the bottom of MDFs coated with PDMS@FDTS-Mg/Al LDH just ignited after 20 s (Figure 8f). The flame height of MDFs coated with PDMS@FDTS-Mg/Al LDH was lower than that of MDFs alone after 60 s (Figure 8g). MDFs coated with PDMS@FDTS-Mg/Al LDH burned out with a white coating resulting from degradation products of Mg/Al LDH (Figure 8h). It was also demonstrated that the PDMS@FDTS-Mg/Al LDH coating improved the flame retardancy of the MDFs.

## 4. Conclusions

In summary, we have successfully fabricated superhydrophobic coatings on MDF with self-cleaning functionalities and flame retardancy. The PDMS@FDTS-Mg/Al LDH coating exhibited superhydrophobicity with a water contact angle of 155° and good household liquid repellency. The self-cleaning test showed that decontamination on the coating can be carried out by flowing water droplets, and muddy water can roll off readily. The LOI value increased from 24.0 (MDFs) to 38.5 (MDFs coated with PDMS@FDTS-Mg/Al LDH). The peak heat release rate and total heat release of MDFs coated with PDMS@FDTS-Mg/Al LDH reduced by 24.7% and 11.2% as compared to MDFs alone. The presented inorganic coating provided a new strategy for the protection and functionalization of MDFs.

## Figures and Tables

**Table 1:** XRD data of Mg/Al LDH and FDTS-Mg/Al LDH.

Samples	003		006		012		015		018		110	
	2θ (°)	d (Å)	2θ (°)	d (Å)	2θ (°)	d (Å)	2θ (°)	d (Å)	2θ (°)	d (Å)	2θ (°)	d (Å)
A ^a^	11.76	7.52	23.58	3.77	34.76	2.58	39.56	2.28	47.24	1.92	60.94	1.52
B ^b^	11.48	7.70	23.16	3.84	34.76	2.58	39.18	2.30	46.64	1.95	60.72	1.53

^a^ A refers to Mg/Al LDH. ^b^ B refers to FDTS-Mg/Al LDH.

**Table 2:** Cone calorimeter data of MDFs and MDFs coated with PDMS@FDTS-Mg/Al LDH.

Samples	TTI ^a^ (s)	PHRR ^a^ (kW/m^2^)	TPHRR ^a^ (s)	FIGRA ^a^ (kW/m^2^ s)	THR ^a^ (MJ/m^2^)
A ^b^	21	298.8	239	1.3	57.1
B ^c^	42	224.9	279	0.8	50.7

^a^ TTI, PHRR, TPHRR, FIGRA, and THR refer to the time to ignition, peak heat release rate, the time from ignition to achieve PHRR, fire growth rate (acquired through dividing PHRR by TPHRR), total heat release, respectively. ^b^ A refers to MDF. ^c^ B refers to MDF coated with PDMS@FDTS-Mg/Al LDH.
